# Reliability of cyclin A assessment on tissue microarrays in breast cancer compared to conventional histological slides

**DOI:** 10.1038/sj.bjc.6603147

**Published:** 2006-05-02

**Authors:** K Aaltonen, C Ahlin, R-M Amini, L Salonen, M-L Fjällskog, P Heikkilä, H Nevanlinna, C Blomqvist

**Affiliations:** 1 Department of Oncology, Helsinki University Central Hospital, PO Box 180, FIN 00290 HUS, Finland; 2 Department of General Oncology, Örebro University Central Hospital, SE 701 85 Örebro, Sweden; 3 Department of Genetics and Pathology, Uppsala University Hospital, SE 751 85 Uppsala, Sweden; 4 Department of Obstetrics and Gynaecology, Helsinki University Central Hospital, PO Box 700, FIN 00029 HUS, Helsinki, Finland; 5 Department of Oncology, Radiology and Clinical Immunology, Uppsala University Hospital, SE 751 85 Uppsala, Sweden; 6 Department of Pathology, Helsinki University Central Hospital, University of Helsinki, PO Box 21, FIN 00014 Helsinki, Finland

**Keywords:** breast cancer, cyclin A, tissue microarray

## Abstract

Cyclin A has in some studies been associated with poor breast cancer survival, although all studies have not confirmed this. Its prognostic significance in breast cancer needs evaluation in larger studies. Tissue microarray (TMA) technique allows a simultaneous analysis of large amount of tumours on a single microscopic slide. This makes a rapid screening of molecular markers in large amount of tumours possible. Because only a small tissue sample of each tumour is punched on an array, the question has arisen about the representativeness of TMA when studying markers that are expressed in only a small proportion of cells. For this reason, we wanted to compare cyclin A expression on TMA and on traditional large sections. Two breast cancer TMAs were constructed of 200 breast tumours diagnosed between 1997–1998. TMA slides and traditional large section slides of these 200 tumours were stained with cyclin A antibody and analysed by two independent readers. The reproducibility of the two readers’ results was good or even very good, with kappa values 0.71–0.87. The agreement of TMA and large section results was good with kappa value 0.62–0.75. Cyclin A overexpression was significantly (*P*<0.001) associated with oestrogen receptor and progesterone receptor negativity and high grade both on TMA and large sections. Cyclin A overexpression was significantly associated with poor metastasis-free survival both on TMA and large sections. The relative risks for metastasis were similar on TMA and large sections. This study suggests that TMA technique could be useful to study histological correlations and prognostic significance of cyclin A on breast cancer on a large scale.

Breast cancer is a heterogeneous disease. Differences in tumour phenotypes lead to varying aggressiveness and ability to respond to a given treatment. Nowadays, breast tumours are classified not only by their morphological but also by immunohistochemical and molecular genetic characteristics.

Tissue microarray (TMA) technique was developed by Kononen *et al* (1998). This technique allows rapid screening of multiple stainings of large amount of tumours. In TMA technology, tissue cylinders (diameter 0.6 mm) are punched from hundreds of different tumour blocks and brought into a recipient TMA block. Sections of the blocks can then be used in simultaneous analysis of all the tumours on DNA, RNA and protein level. Tissue microarray technique only takes out a small, cylindrical specimen from the donor block. This minimises the tissue damage to the donor block and allows its use in many studies, but still leaves a virtually undamaged tissue block for the pathologist.

In TMA, only a small amount of tumour (0.6 mm) is analysed, leading to the question of how representative the minute tissue on TMA is and how much tumour heterogeneity affects the results. Many studies have shown that although a result of an individual tumour on TMA and on a large section may vary, the correlation to histopathological factors and prognostic implications are similar when large numbers of tumours are studied (Kononen *et al*, 1998; Camp *et al*, 2000; Gillett *et al*, 2000; Nocito *et al*, 2001; Torhorst *et al*, 2001). In breast cancer, the expression of known prognostic and predictive factors such ER (oestrogen receptor), PR (progesterone receptor) and HER2 (human epidermal growth factor receptor 2) were reliably analysed on TMA (Kononen *et al*, 1998; Camp *et al*, 2000; Gillett *et al*, 2000; Torhorst *et al*, 2001), but it is still unclear as to how representative TMA is when molecules that show focal localisation within the tumour are studied (Gillett *et al*, 2000).

More than 80% of breast cancer patients are alive 5 years after the initial diagnosis (Finnish Cancer Registry, 2002). The best established prognostic factors are tumour size and the number of the involved regional lymph nodes (Singletary *et al*, 2003). Grade of the tumour, hormone receptor (ER, PR) status and proliferation rate are also well-documented prognostic factors, especially in node-negative disease (Ross and Fletcher, 1999; Schnitt, 2001). Of these factors, only ER, PR and proliferation rate predict treatment efficacy. Expression of ER or PR is a prerequisite for endocrine responsiveness (EBCTCG, 2005), whereas a high proliferation rate predicts a more favourable chemotherapy response (Hietanen *et al*, 1995).

The best documented method of measuring proliferation in breast cancer is SPF (S-phase fraction) assessed by flow-cytometry (Sigurdsson *et al*, 1990). The method is, however, cumbersome and requires fresh tissue. In recent years, immunohistochemistry has gained popularity for the assessment of proliferation. Ki-67 is the most studied proliferation antigen, but there still is no consensus on methodology or cutoff values for prognostification in breast cancer (Colozza *et al*, 2005). Cyclins are cell cycle regulators overexpressed in several tumours including breast cancer (Parwaresch and Rudolph, 1996). Cyclin A is required for replication during the S-phase and is also expressed in the early mitotic phase (G2) of the cell cycle controlling the cell entry into mitosis (Parwaresch and Rudolph, 1996). In three previous studies in sarcomas and head and neck cancer, cyclin A was associated with prognosis and chemotherapy response even stronger than Ki-67 (Huuhtanen *et al*, 1999a, 1999b; Saarilahti *et al*, 2003). Several studies have shown an association between overexpression of cyclin A and poor survival in breast cancer (Bukholm *et al*, 2003a, 2003b, 2001; Michalides *et al*, 2002; Michels *et al*, 2003), whereas one study did not confirm it (Kuhling *et al*, 2003; Rudolph *et al*, 2003). Further studies with a larger number of patients are needed, including also specific groups of patients like those with hereditary predisposition to breast cancer. Tissue microarray seems a tempting method to evaluate cyclin A expression on breast tumours. Cyclins D1 and E have been studied on TMA (Richter *et al*, 2000; Han *et al*, 2003; Hedberg *et al*, 2003; Jirstrom *et al*, 2003; Schraml *et al*, 2003; Stendahl *et al*, 2004). However, cyclin A has not yet been analysed on breast cancer TMA. In this study, we evaluated cyclin A expression of 200 breast tumours on TMA and large sections in order to evaluate the representativity of TMA for the assessment of proliferation compared to large sections.

## MATERIALS AND METHODS

### Patients

Tumours from 200 breast cancer patients, treated in Helsinki University Central Hospital between 1997 and 1998 (Syrjakoski *et al*, 2000), were analysed on TMA and traditional large sections. Pathology reports of the primary tumours were studied. Pathological data including information on tumour histology, grade, ER and PR status, tumour diameter, nodal status and distant metastases were collected. Grading was performed according to Scarff–Bloom–Richardson modified by Elston and Ellis (1991). Patients’ records were studied and information on adjuvant treatment, local relapse and distant metastases as well as time of death or follow-up were collected. Patient characteristics are shown in Table 1.

### Tissue microarray construction

Paraffin blocks of primary tumours of the patients were collected. Haematoxylin and eosin sections of the original blocks were studied and the most representative area of each tumour was punched and brought into two recipient paraffin blocks to produce two breast cancer microarrays, including four cores (diameter 0.6 mm) of each tumour. Then, 3- to 4-*μ*m- thick sections were then cut from array blocks and transferred to glass slide (Eerola *et al*, 2005; Tommiska *et al*, 2005).

### Immunohistochemistry

Formalin-fixed paraffin-embedded tissue material from the blocks used for TMA sampling from each of the 200 tumours and their corresponding TMA slides were cut in 3- to 4-*μ*m-thick sections and deparaffinised. Immunostaining for cyclin A (mouse monoclonal; Novo Castra Laboratories) was carried out manually. Antigen retrieval was carried out using a pressure cooker for 5 min in 0.01 M citrate buffer, pH 6.0. Primary antibody was diluted 1 : 300 and applied for overnight incubation. Staining was carried out using the avidin–biotin peroxidase complex and AEC procedures (Wood and Warnke, 1981). The peroxidase was developed using the DAB technique.

Two readers (KA and CA) scored all slides. The percentage of cyclin A-positive breast cancer cells was counted in one high-power field ( × 40 objective) in each of the four tissue cores on TMA and in three randomly selected and one ‘hot-spot’ high-power field on large sections. A minimum of 200 breast cancer cells and in most cases at least 400 breast cancer cells were counted from each tumour. To better demonstrate the pathological associations, results were dichotomised at a cutoff value of 10% (Poikonen *et al*, 2005). All statistical evaluations were carried out with both average and maximal values of the four counts.

### Statistical analysis

All statistical analyses were made using Macintosh SPSS 11 statistical software package. Correlations of TMA and large section results as well as correlations of two independent readers’ results were evaluated by crosstabs and by a scatter diagram showing the differences of the results plotted against the average of the results. Kappa values were counted to evaluate the reproducibility of the results by two readers and by two different methods. Student's *t*-test for independent samples was used to analyse whether disconcordant results on large section and TMA or between the two readers was owing to a low number of cells counted. Correlation of cyclin A expression to ER, PR, nodal status and grade were analysed by the nonparametric Spearman's correlation coefficient. All statistical tests of the association of cyclin A and clinical parameters as well as survival analysis with the Cox proportional-hazard model were performed with cyclin A percentage as a continuous variable. To improve readability, relative risks for metastasis or death was calculated for cyclin A dichotomised at a cutoff of 10%.

## RESULTS

On TMA, results of 14 tumours (7%) were missing, five of them owing to loss of all four punches during the staining and nine because of lack of tumour in the arrayed samples. On large sections, result of only one tumour (0.5%) was missing, and the reason was unsuccessful staining. The median cyclin A count 3.7% (range 0–34.4%) on TMA average values, 5.8% (0–52.9%) on TMA maximum values, 4.3% (0–32.1%) on large section average values and 9.0% (0–39.1%) on large section maximum values.

### Agreement of readers’ results

**Figure 1:**
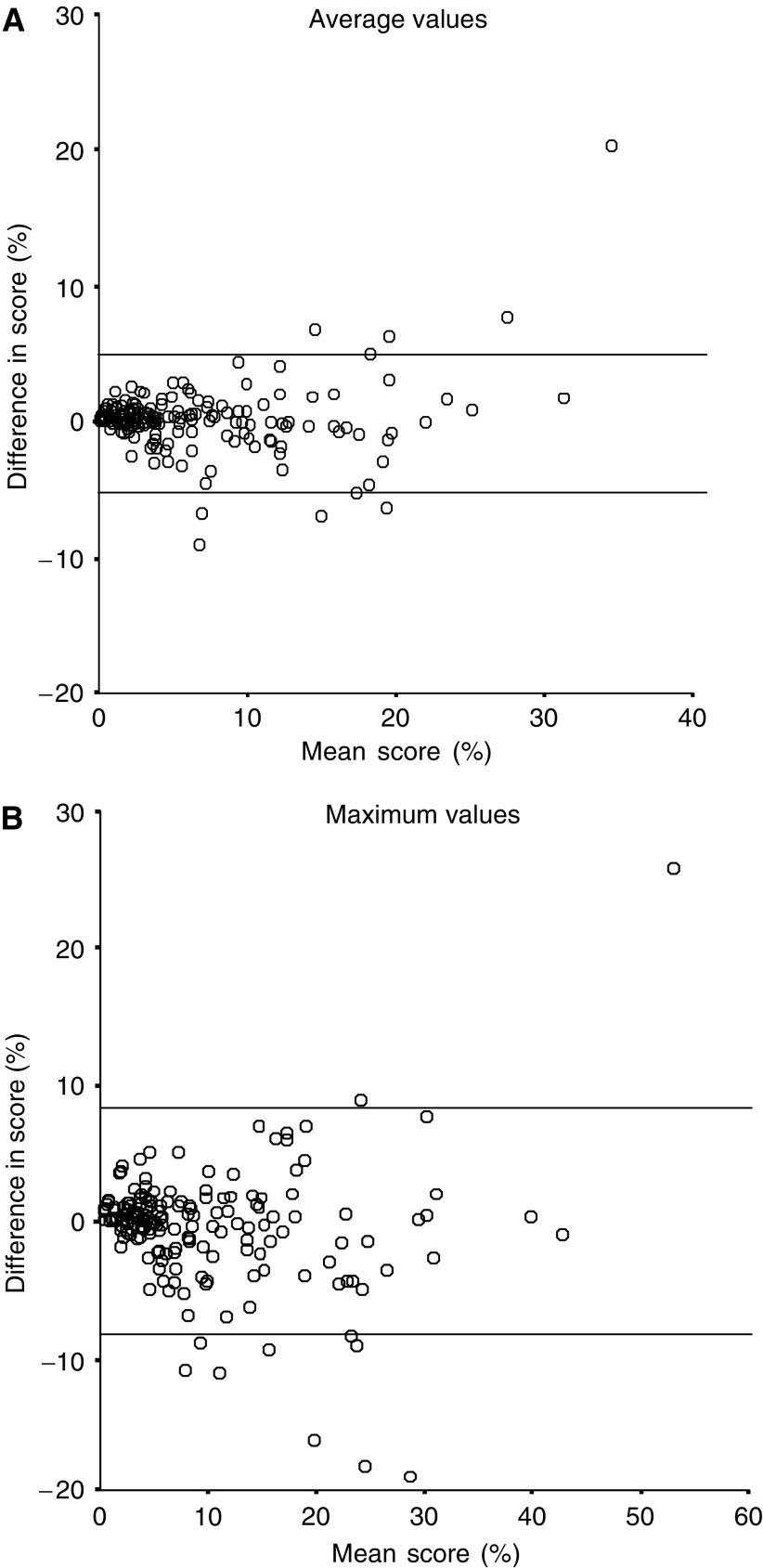
(**A**) Scattergram shows the difference in two readers score (KA−CA) compared to mean score of the two readers (KA+CA/2) on TMA average values. Lines indicate the 95% limits of agreement. (**B**) Scattergram shows the difference in two readers score (KA−CA) compared to mean score of the two readers (KA+CA/2) TMA maximum values. Lines indicate the 95% limits of agreement.

**Figure 2:**
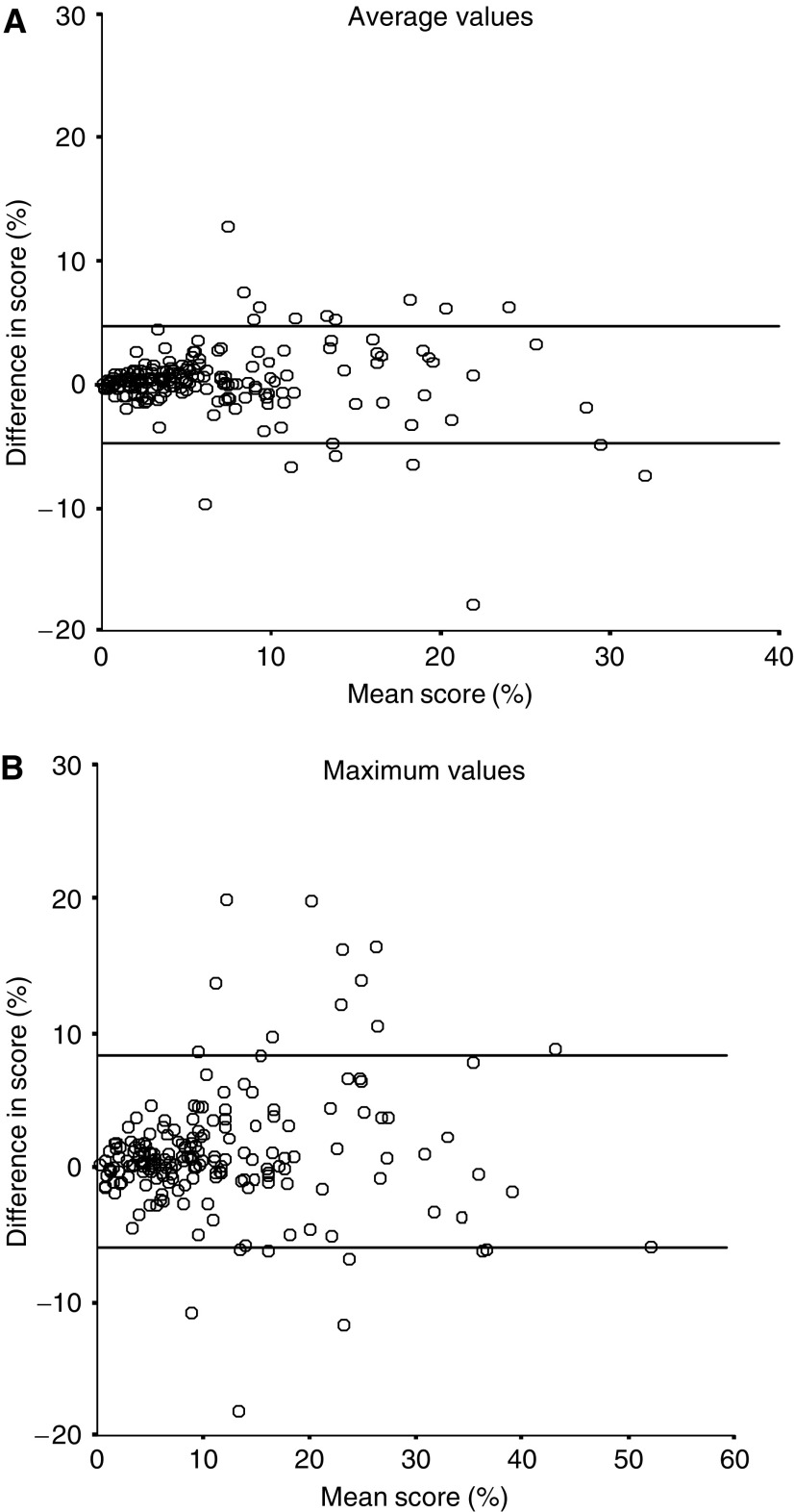
(**A**) Scattergram shows the difference in two readers score (KA−CA) compared to mean score of the two readers (KA+CA/2) on large section average values. Lines indicate the 95% limits of agreement. (**B**) Scattergram shows the difference in two readers score (KA−CA) compared to mean score of the two readers (KA+CA/2) on large section maximum values. Lines indicate the 95% limits of agreement.

Figures 1 and 2 show scatter diagrams of the differences of two readers’ results. The mean difference between the two readers scoring and 95% limits of agreement were 0.1% (−4.8 to +5.1%) for TMA average values, 0.4% (−8.0 to +8.8%) for TMA maximum values, 0.4% (−4.4 to +5.1%) for large section average values and 1.0% (−7.5 to +9.5%) for large section maximum values. The kappa values evaluating the reproducibility two readers’ results were 0.87 for TMA average value, 0.83 for TMA maximum value, 0.71 for large section average value and 0.80 for large section maximum value. The concordance of the positive and the negative results for the two readers with 10% as a cutoff point is shown in Table 2. In order to evaluate possible reasons for discrepant results of the two readers, we calculated the mean number of nuclei counted for concordant *vs* discrepant cases. The mean number is the mean of the two readers results. The mean number of nuclei counted was 661 in concordant tumours and 420 in discrepant tumours (*P*=0.002) for array average values, 671 in concordant tumours and 410 in discrepant tumours (*P*<0.0005) for array maximum values, 767 in concordant tumours and 465 in discrepant tumours (*P*<0.0005) for large section average values and 759 in concordant tumours and 518 in discrepant tumours for large section maximum values (*P*<0.0005).

### Agreement of TMA and large sections on cyclin A staining

**Figure 3:**
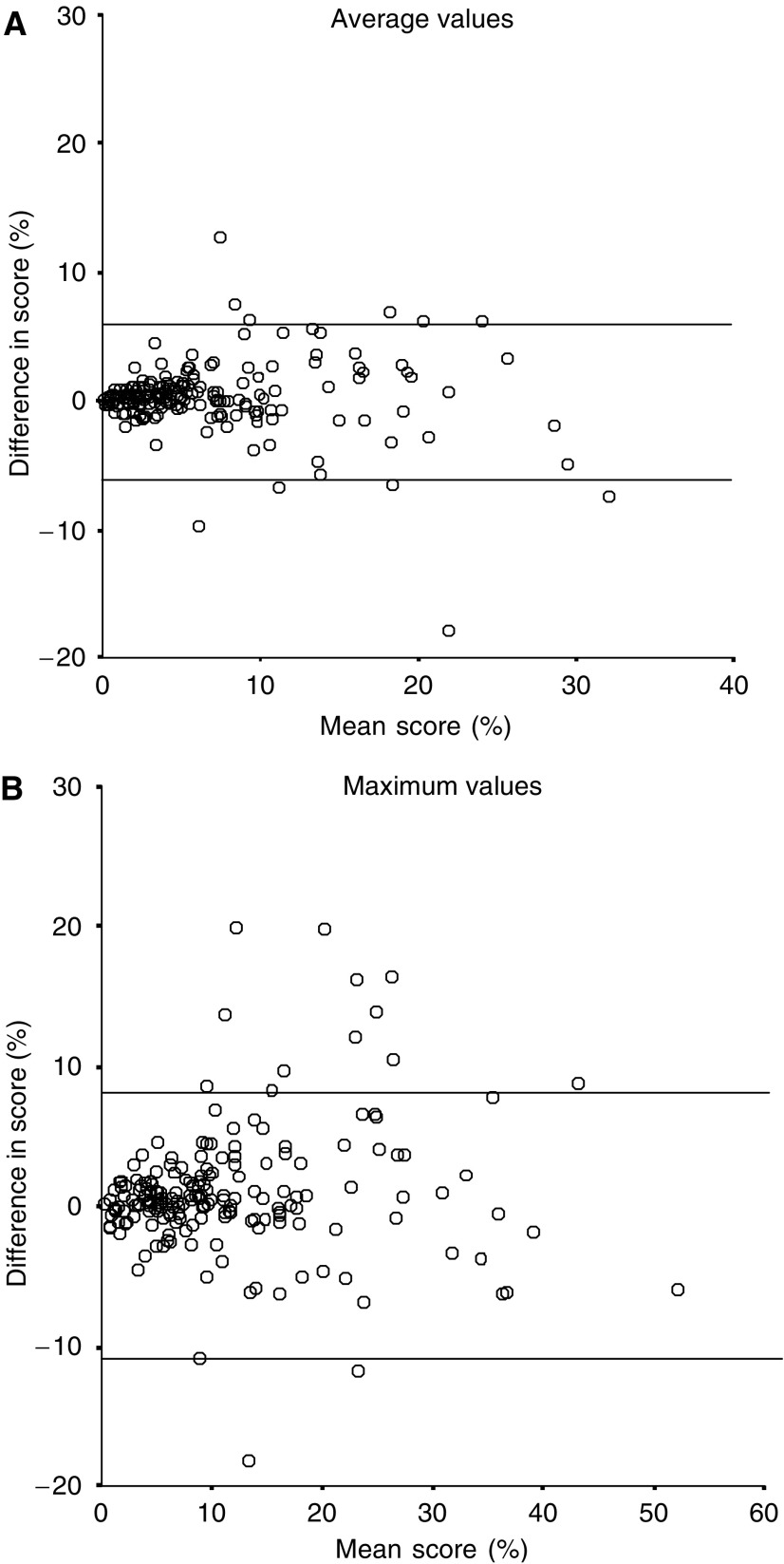
(**A**) Scattergram shows the difference in array and large section scores (array−large section) compared to large section score on average values. Lines indicate the 95% limits of agreement. (**B**) Scattergram shows the difference in array and large section scores (array−large section) compared to large section score on maximum values. Lines indicate the 95% limits of agreement.

Tissue microarray and large section cyclin A results were compared using the average values of the two readers’ results. Figure 3 shows a scatter diagram of the differences of TMA and large section results. The mean difference between the TMA and large section scoring and 95% limits of agreement were 0.4% (−6.9 to +7.6%) for average values and 2.0% (−8.7 to +12.6%) for maximum values. The kappa values were 0.75 for average values and 0.62 for maximum values. The agreement for classification of a high cyclin A score between TMA and sections is shown in Table 3. The mean amount of nuclei counted was 683 in concordant tumours and 308 in discrepant tumours for average values (*P*<0.0005). For maximum values, the mean amount of cells counted in concordant tumours was 661 and 612 in discrepant tumours (*P*=0.33).

When associations of cyclin A and histological variables were studied, the results on TMA and large sections were well in concordance. High cyclin A expression associated strongly with ER negativity (*P*<0.0005), PR negativity (*P*<0.0005) and high grade (*P*<0.0005). The associations were similar when cyclin A expression was analysed with TMA maximum value, TMA average value, large section maximum value and large section average value (Table 4). Cyclin A expression was not associated with nodal status of the tumour.

Cyclin A overexpression was not statistically significantly associated with overall survival; however, it was significantly associated with poorer metastasis-free survival (Table 5).

## DISCUSSION

In this study, we investigated cyclin A expression of 200 breast tumours on TMA and on traditional large sections and we found that the agreement between these methods is good, as well as the reproducibility of the results by two independent readers. The main aim of this study with a relatively small patient population of 200 patients was to evaluate the suitability of TMA analysis for a larger scale study on the prognostic significance of cyclin A and not to clarify the prognostic value of cyclin A overexpression. All histological correlations, however, were similar on TMA and large section. The results suggest that relative risks for overall and metastasis-free survival can be evaluated on TMA as reliably as on large sections.

To evaluate the reproducibility of cyclin A results, we first compared the agreement of two readers’ results. The kappa value is considered as a reliable tool to assess this agreement (Altman, 1991). The agreement is good if the kappa value is 0.61–0.80 and very good if the kappa value is 0.81–1.00 (Altman, 1991). We found the reproducibility of cyclin A assessment for the two readers to be good or very good both in large sections and TMAs. As expected, the assessment of maximum proliferation rate using hot-spots was less reproducible. One important reason for disagreement seems to be the amount of tissues studied, as discrepant findings seemed to be associated with low number of nuclei counted. In a recent study, the reproducibility of malignancy grading was only moderately good (Meyer *et al*, 2005), suggesting that measurement of proliferation by immunohistochemically detectable markers will probably give superior results. Our study shows that reproducibility of cyclin A expression on TMA is good and it seems to be a method that could easily be adapted to routine use.

As the results of two readers were in concordance, we counted the average of the results of two readers and used these results to analyse the concordance of TMA and large section results. The agreement between cyclin A assessment on TMAs and large slides was good for both mean and maximum value, but as expected somewhat better for the former. Again, the average results that differ most from each other were results of tumours with fewer cells counted. The agreement was moderately weaker on maximum than average values. One reason for this could be the method used in counting cells: on TMA, one randomly selected high-power field was counted on each punch, but on large sections, we searched one ‘hot-spot’ high-power field and selected the other three randomly. Earlier studies in breast cancer TMA have shown good agreement of ER, PR, p53 and HER2 between TMA and large sections (Camp *et al*, 2000; Gillett *et al*, 2000; Torhorst *et al*, 2001). Expression of cyclins E and D1 were in concordance in 94.3 and 95.4% of cases, respectively, in a previous study of 175 breast tumours (Han *et al*, 2003). The proliferation marker Ki-67 was studied in a previous bladder carcinoma study comprising 2317 cases and the concordance of TMA and large sections was good (Nocito *et al*, 2001). Cyclin E expression on TMA and large section has also been evaluated in a series of 218 renal cell carcinomas and the agreement was good (*P*<0.001) (Hedberg *et al*, 2003).

Cyclin A overexpression was associated with ER and PR negativity and with high grade, in line with earlier studies (Michalides *et al*, 2002; Michels *et al*, 2003). A high cyclin A score was also prognostic for distant recurrence both in large sections and TMAs. Most previous studies have shown association between cyclin A overexpression and poor prognosis (Bukholm *et al*, 2001, 2003a; Michalides *et al*, 2002; Michels *et al*, 2003), whereas one study failed to find an association (Kuhling *et al*, 2003; Rudolph *et al*, 2003). Thus in concordance with other studies in various malignancies, this study confirms that TMAs are well suited for assessment of immunohistochemical markers even for antigens with a variable expression in different parts of a tumour. The similarity of clinicopathological associations on TMAs and large sections also support the validity of TMA for scoring of cyclin A in breast cancer.

Considering our results, we conclude that TMA is as good as large sections in scoring for cyclin A on breast cancer.

## Figures and Tables

**Table 1:** Patient characteristics

*Grade*		*T*	
1	47 (23.5%)	1	111 (55.5%)
2	86 (43.0%)	2	71 (35.5%)
3	58 (29.0%)	3	6 (3.0%)
Not known	9 (4.5%)	4	7 (3.5%)
		Not known	5 (2.5%)
			
*N*		*M*	
Node positive	96 (48.0%)	0	190 (95.0%)
Node negative	98 (49.0%)	1	9 (4.5%)
Not known	6 (3.0%)	Not known	1 (0.5%)
			
*Oestrogen receptor*		*Progesterone receptor*	
Positive	155 (77.5%)	Positive	137 (68.5%)
Negative	35 (17.5%)	Negative	53 (26.5%)
Not known	10 (5.0%)	Not known	10 (5.0%)
			
*Local relapse*		*Distant metastases*	
Yes	22 (11.0%)	Yes	49 (24.5%)
No	178 (89.0%)	No	151 (75.5%)
			
*Adjuvant radiotherapy*		*Adjuvant chemotherapy*	
Yes	173 (86.5%)	Yes	78 (39.0%)
No	27 (13.5%)	No	122 (61.0%)
			
*Adjuvant hormone therapy*			
Yes	91 (45.5%)		
No	109 (54.5%)		

**Table 2:** Comparison of the results of two independent readers

*TMA average results*		
Positive cells	Negative cells	
37 (20.4%)	3 (1.7%)	Positive cells
5 (2.7%)	136 (75.1%)	Negative cells
		
*TMA maximum results*		
Positive cells	Negative cells	
57 (31.4%)	3 (1.7%)	Positive cells
11 (6.1%)	110 (60.8%)	Negative cells
		
*Large section average results*		
Positive cells	Negative cells	
31 (15.6%)	10 (5.0%)	Positive cells
10 (5.0%)	148 (74.4%)	Negative cells
		
*Large section maximum results*		
Positive cells	Negative cells	
74 (37.2%)	14 (7.0%)	Positive cells
5 (2.5%)	106 (53.3%)	Negative cells

TMA=tissue microarray.

**Table 3:** Comparison of tissue microarray and large section results

*Average results*		
Array, positive cells	Array, negative cells	
30 (16.1%)	8 (4.3%)	Large section, positive cells
7 (3.8%)	141 (75.8%)	Large section, negative cells
		
*Maximum results*		
Array, positive cells	Array, negative cells	
53 (28.5%)	27 (14.5%)	Large section, positive cells
7 (3.8%)	99 (53.2%)	Large section, negative cells

**Table 4:** Cyclin A and correlation to histological variables

	**Spearman's correlation coefficient**	***P*-value**
*ER*		
TMA average value	−0.417	<0.0005
TMA maximum value	−0.414	<0.0005
Large section average value	−0.453	<0.0005
Large section maximum value	−0.459	<0.0005
		
*PR*		
TMA average value	−0.427	<0.0005
TMA maximum value	−0.427	<0.0005
Large section average value	−0.474	<0.0005
Large section maximum value	−0.450	<0.0005
		
*Grade*		
TMA average value	0.529	<0.0005
TMA maximum value	0.537	<0.0005
Large section average value	0.555	<0.0005
Large section maximum value	0.523	<0.0005
		
*Nodal status*		
TMA average value	−0.078	0.295
TMA maximum value	−0.065	0.384
Large section average value	−0.046	0.539
Large section maximum value	−0.023	0.762

TMA=tissue microarray.

**Table 5:** Survival and cyclin A overexpression

	**Hazard for low *vs* high cyclin A^a^**	***P*-value^b^**
*(a) Overall survival*		
TMA average result	1.338	0.128
Large section average result	1.205	0.639
TMA maximum result	1.651	0.173
Large section maximum result	1.393	0.755
		
*(b) Overall survival in multivariate analysis with adjuvant chemotherapy and hormone therapy*		
TMA average result	1.580	0.051
Large section average result	1.540	0.503
TMA maximum result	1.915	0.076
Large section maximum result	1.194	0.534
		
*(c) Metastasis free survival*		
TMA average result	1.765	0.026
Large section average result	1.831	0.117
TMA maximum result	1.858	0.022
Large section maximum result	1.463	0.021
		
*(d) Metastasis free survival in multivariate analysis with adjuvant chemotherapy and hormone therapy*		
TMA average result	2.100	0.009
Large section average result	1.950	0.075
TMA maximum result	2.149	0.009
Large section maximum result	1.528	0.019

a Low/high cut off value 10%.

b Cyclin A analyzed as a continuous variable.
